# Putative chemosensory receptors are differentially expressed in the sensory organs of male and female crown-of-thorns starfish, *Acanthaster planci*

**DOI:** 10.1186/s12864-018-5246-0

**Published:** 2018-11-29

**Authors:** R. E. Roberts, D. Powell, T. Wang, M. H. Hall, C. A. Motti, S. F. Cummins

**Affiliations:** 1 Genecology Research Centre, Faculty of Science, Health, Education and Engineering, University of the Sunshine Coast, Maroochydore DC, QLD 4558 Australia; 2 Australian Institute of Marine Science (AIMS), Cape Ferguson, Townsville, QLD 4810 Australia

**Keywords:** Chemosensation, Olfaction, GPCR, IR, Starfish, COTS, RNASeq, Differential gene expression

## Abstract

**Background:**

Chemosensation is a critical signalling process for all organisms and is achieved through the interaction between chemosensory receptors and their ligands. The Crown-of-thorns starfish, *Acanthaster planci* species complex (COTS), is a predator of coral polyps and *Acanthaster* cf. *solaris* is currently considered to be one of the main drivers of coral loss on the Great Barrier Reef in Queensland, Australia.

**Results:**

This study reveals the presence of putative variant Ionotropic Receptors (IRs) which are differentially expressed in the olfactory organs of COTS. Several other types of G protein-coupled receptors such as adrenergic, metabotropic glutamate, cholecystokinin, trace-amine associated, GRL101 and GPCR52 receptors have also been identified. Several receptors display male-biased expression within the sensory tentacles, indicating possible reproductive significance.

**Conclusions:**

Many of the receptors identified in this study may have a role in reproduction and are therefore key targets for further investigation. Based on their differential expression within the olfactory organs and presence in multiple tissues, it is possible that several of these receptor types have expanded within the Echinoderm lineage. Many are likely to be species-specific with novel ligand-binding affinity and a diverse range of functions. This study is the first to describe the presence of variant Ionotropic Glutamate Receptors in any Echinoderm, and is only the second study to investigate chemosensory receptors in any starfish or marine pest. These results represent a significant step forward in understanding the chemosensory abilities of COTS.

**Electronic supplementary material:**

The online version of this article (10.1186/s12864-018-5246-0) contains supplementary material, which is available to authorized users.

## Background

Chemosensation is a critical signalling process for all organisms and is achieved through the interaction between chemosensory receptors and their ligands [1]. This process is particularly important in phyla which may lack other well-developed senses, including many invertebrate phyla. Olfactory receptors (ORs) are typically G protein-coupled receptors (GPCRs), however, many species also utilise other receptor types for chemosensory signalling. For example, *Drosophila* were the first organisms discovered to utilise Ionotropic Receptors (IRs) for olfaction [1]. More recently, similar IRs have been characterised in a wide variety of organisms including fish [2], molluscs [3], crustaceans [4] and other insects such as Lepidopterans [5].

Amongst the complexity of environmental molecules, ORs can be specific to pheromones, which are crucial for regulating mate attraction in many species from invertebrates to mammals [6]. Pheromone receptors and their corresponding ligands are often differentially expressed in specific tissues between the males and females of the same species. Female moths, for instance, secrete pheromone molecules which can only be detected by male conspecifics through specific receptors [7]. As a general rule, chemosensory receptors such as ORs and gustatory receptors (GRs) are expressed in the sensory epithelia of specialised organs, however, there is a growing body of evidence across multiple phyla to suggest that chemosensory receptors may be expressed and functional in a variety of other tissues [8, 9]. While there is an extensive base of knowledge about olfaction in terrestrial species, comparatively little is known about these processes in aquatic organisms.

In the last decade, advances in high-throughput sequencing technology has not only enabled the sequencing of whole genomes and transcriptomes, but has also made it possible to detect minute differences in gene expression levels between individuals, genders, tissues, and life stages of organisms via quantitative RNA-seq [10]. A plethora of recent studies have utilised an RNA-seq approach to investigate ORs in insects, providing a foundation for novel pest management approaches within the agricultural industry. This includes identification of ORs in the Light brown apple moth, *Epiphyas postvittana* [11], the White-backed planthopper, *Sogatella furcifera* [12], the Codling moth, *Cydia pomonella* [13] and the Asian longhorned beetle *Anoplophora glabripennis* [14], all of which are pests that cause significant crop damage in agriculture worldwide. RNA-seq has also been used to identify differentially expressed ORs in various life stages of the wild Atlantic salmon, *Salmo salar*, including those which are critical for homing and migration [15]. Despite these significant advances and the obvious importance of identifying ORs in pest species, this avenue has not been explored in any marine pests.

The Crown-of-thorns starfish, *Acanthaster planci* species complex (COTS), is a predator of coral polyps and causes significant damage to coral reefs worldwide [16]. *Acanthaster* cf. *solaris* [17] is currently considered to be one of the main drivers of coral loss in the Indo-Pacific [18, 19]. This species complex experiences cyclic population explosions where entire reefs can be decimated, taking decades to recover. Current technologies have enabled sequencing of the genomes of non-model organisms, including echinoderms such as the Japanese spiky sea cucumber, *Apostichopus japonicas* [20] and the purple sea urchin, *Strongylocentrotus purpuratus* [21]. The recent publication of the COTS genome [19] has facilitated the investigation of the molecular mechanisms underpinning COTS’ behaviours, including the identification of ‘*Acanthaster planci* putative ORs’ (ApORs) [22]. ApORs were first discovered in the transcriptomes of two putative olfactory organs, the tube feet (TF) and sensory tentacle (ST). These organs form the peripheral portion of the water vascular system and are external hydraulic structures; the TF extend along each arm of the starfish on the posterior surface, and the STs are found at the tip of each arm. It has long been assumed that STs contain a higher proportion of chemosensory receptors than TF, hence their name [23]. Supporting this hypothesis is the discovery of more ApORs in the transcriptome of STs than that of TF [22]. However, it is still unknown whether olfaction in COTS is predominantly isolated to its TF and STs, or if ApORs are also expressed in other tissues, as has been discovered in many other species [24].

Given the recent discovery of a large family of 63 putative ApORs in COTS [22], a key issue is to refine this extensive list to identify a practical number for further testing in functional bioassays. Therefore, the main objective of this study was to identify differentially expressed putative chemosensory genes between COTS TF and STs in both males and females, via an RNA-seq quantitative approach, and then to specifically examine the differential gene expression profiles of the 63 previously identified ApORs.

## Methods

### COTS sample collection and RNA-seq

COTS were obtained from outbreak-affected regions of the Great Barrier Reef off the coast of Cairns, Queensland, from 21 to 26 June, 2016, during the regenerative period of the COTS reproductive cycle [25]. To ensure sexual maturity and to minimise size-related variation, all tissue samples used in this study were collected from individuals ranging from 300 to 400 mm in diameter. After collection, animals were kept in tanks on board the research vessel no longer than 2 h before dissection. Sex of individuals was determined by gonad visualisation. Samples were taken from three male and three female COTS for three biological replicates of each tissue. Tube foot and sensory tentacle tissues were dissected and placed immediately in RNA*later* solution (Life Technologies) before transportation to the laboratory at the University of the Sunshine Coast, Maroochydore, where they were subsequently frozen at − 80 °C.

Three biological replicates each of TF and ST were taken from both male and female COTS, resulting in four experimental groups: male ST (*n* = 3), female ST (n = 3), male TF (n = 3) and female TF (n = 3). Tissue samples were thawed and weighed before homogenisation in Trizol reagent (Invitrogen) and total RNA extraction using the Direct-zol RNA MiniPrep Plus kit (Zymo Research). RNA concentration was determined via Nanodrop spectrophotometer (Thermo Scientific). Approximately 1 μg total RNA from each sample was sent to the Australian Genomic Research Facility (Melbourne, Australia). Library preparation was done using the illumina TruSeq RNA library prep kit with poly A enrichment and the Illumina HiSeq 2500 platform was used to generate 100 base-pair single-end reads.

### Read mapping and identification of differentially expressed gene transcripts in COTS

RNAseq analysis was conducted following the general approach of Trapnell et al. [26]. Reference COTS genome and annotation files were obtained from the OIST Genome Browser (http://marinegenomics.oist.jp) [19]. FastQC was used to assess quality of raw reads (http://www.bioinformatics.babraham.ac.uk/projects/fastqc/). Adaptors and low-quality reads were filtered using Trimmomatic v.0.32 [27]. The reference genome index was constructed with Bowtie v.2.0.6 [28] and clean reads were aligned to the reference genome using TopHat v.2.0.9 [26]. Aligned reads were then assembled using Cufflinks v.2.1.1 [26] and guided by the COTS gene models before count tables for each transcript were generated using HTSeq [29]. Differential gene expression was determined by comparing three sets of experimental groups: i) female ST vs male ST (ST_F_ vs. ST_M_), ii) female TF vs male tube TF (TF_F_ vs. TF_M_) and iii) male/female TF vs male/female STs (TF_ALL_ vs. ST_ALL_). Differentially expressed transcripts were identified using the Bioconductor package DESeq2 in R [30]. Transcripts were considered significantly differentially expressed when they had a false discovery rate (FDR)-corrected *P*-value of < 0.05. Differentially expressed genes (DEGs) were then annotated using BLASTp against the GenBank non-redundant database using an *E*-value threshold of 1*E*-05. Gene ontologies were assigned using BLAST2GO [31]. Differentially expressed genes within the cut-offs with the keyword ‘receptor’ in the BLAST annotation were then used for further analysis, and 11 of these were selected for validation via RT-PCR. ApOR genes previously identified by Roberts et al. [22] were subjected to BLASTp against the DEGs, using an *E*-value threshold of 0 and an identity cut-off of ≥98%. Expression of DEGs in other COTS tissues were investigated using the publicly available transcriptomes from the COTS genome browser (http://marinegenomics.oist.jp) [19]. This comprised of male TF (TF_M_), male mouth (MO_M_), male spine (SP_M_), male radial nerve (RN_M_), female radial nerve (RN_F_), testis, oocyte, early gastrula (EG), mid gastrula (MG). Also included were the female tube foot (TF_F_) and sensory tentacle (ST_F_) transcriptomes published by Roberts et al. [22].

### Multiple sequence alignment, phylogenetic analysis and protein structure visualisation

Homologs for each of the 11 differentially expressed genes in other phyla were curated from the NCBI protein database. Variant ionotropic receptors and ionotropic glutamate receptors from *Aplysia californica* and other phyla were obtained from the supplementary data of Croset et al. (2010). *Strongylocentrotus purpuratus* adrenergic receptors were obtained from Echinobase (http://www.echinobase.org/Echinobase/). Multiple sequence alignments were performed with the Muscle algorithm in the Molecular Evolutionary Genetic Analysis (MEGA) version 7 software [32]. Phylogenies were generated in MEGA using either the neighbour joining or maximum likelihood method with 1000 bootstrap replicates. Protein structures were visualised using Protter v.1.0 [33].

### Tissue-specific gene expression in COTS using RT-PCR

Several COTS obtained from outbreak-affected regions of the Great Barrier Reef off the coast of Cairns, Queensland, were transported live to the Australian Institute of Marine Science in Townsville. Putative chemosensory tissues including TF, ST, radial nerve (RN), cardiac stomach (CS) and body wall (BW) were collected from two male and two female COTS (established by gonad visualisation) and placed immediately in RNA*later* solution (Life Technologies) before being transported to the University of the Sunshine Coast where they were subsequently frozen at − 80 °C. Tissues were thawed and weighed before homogenisation using TriZol reagent (Life Technologies) and subsequent total RNA isolation following manufacturer’s instructions. Following isolation, RNA was assessed for quality by visualisation on a 1.2% agarose gel, and quantified using a Nanodrop spectrophotometer (Thermo Scientific). For each tissue type, male samples were pooled and female samples were pooled. Genomic DNA contamination was removed and first-strand cDNA was synthesised from 1 μg total RNA using random hexamers and the QuantiTect Reverse Transcription Kit (Qiagen). Gene-specific primers were designed from the transcriptome-derived nucleotide sequences using Primer3 v.0.4.0 [34] (Additional file 1: **Table S1**). RT-PCR was conducted using Platinum Hot Start Taq Master Mix (Invitrogen) with 2 μl cDNA template. Cycling parameters were as follows: initial denaturation at 94 °C for 2 min, then 40 cycles of denaturation at 94 °C for 30 s, annealing at 55 °C for 30 s and extension at 72 °C for 1 min. PCR products were analysed via electrophoresis on a 1% agarose gel stained with ethidium bromide. Actin was amplified from the same cDNA template as a positive control and negative control used no template.

## Results

**Fig. 1:**
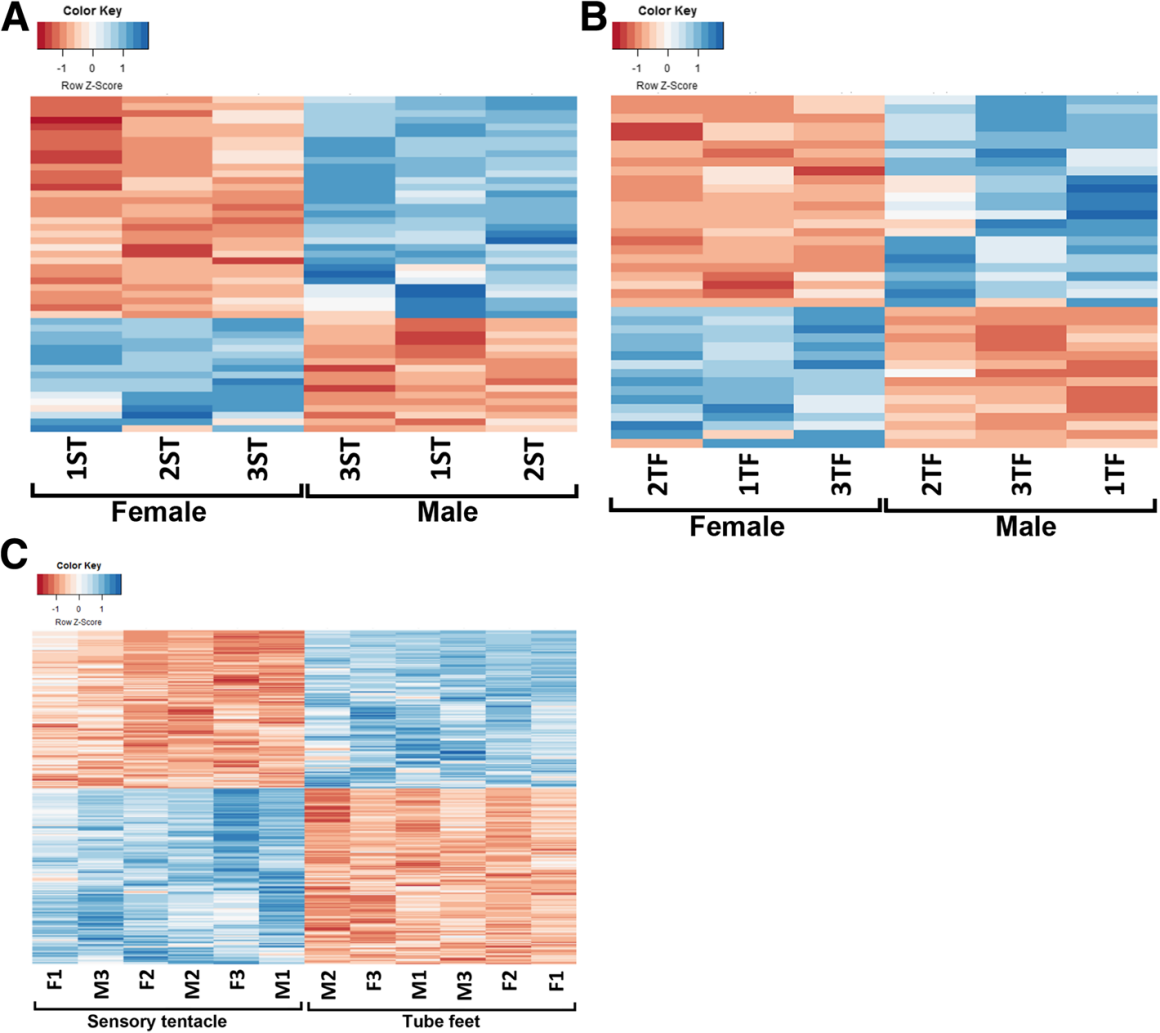
Heatmaps showing differential gene expression in comparisons 1 to 3. **a** Female ST versus male ST (ST_F_ vs. ST_M_). **b** Female TF versus male TF (TF_F_ vs. TF_M_). **c** Combined male/female ST versus combined male/female TF (TF_ALL_ vs. ST_ALL_)

Following mapping, the combined COTS sensory tentacle and tube feet transcriptomes yielded 69,342 assembled contigs with a mean length of 3151 base pairs (bp) and N50 of 5431 bp. Differential gene expression analysis was then performed on the three sets of experimental groups (Additional file 2: **File S1a-c**), and hierarchical clustering placed samples into distinct clusters based on their level of DEG and condition (Fig. 1). Set 1, consisting of female ST versus male ST (ST_F_ vs. ST_M_) displayed 59 significantly DEGs (Fig. 1a); 20 were overexpressed in ST_M_, and 39 were overexpressed in ST_F_. Six of the DEGs in this comparison had a BLAST annotation containing the keyword ‘receptor’, five were overexpressed in ST_M_ and the other was overexpressed in ST_F_.

Set 2, consisting of female TF versus male TF (TF_F_ vs. TF_M_), contained 30 significantly DEGs (Fig. 1b); 13 were overexpressed in TF_M_ and 17 were overexpressed in TF_F_. One of the DEGs in this group contained the keyword ‘receptor’ and was overexpressed in TF_F_. Set 3, consisting of combined male/female ST versus combined male/female TF (TF_ALL_ vs. ST_ALL_) had the largest number of significantly DEGs, at 2583 (Fig. 1c); 1171 were overexpressed in ST_ALL_ and 1412 were overexpressed in TF_ALL_. Of this comparison 295 significantly DEGs contained the keyword ‘receptor’ 208 of which were overexpressed in ST_ALL_ and 79 in TF_ALL_.

**Fig. 2:**
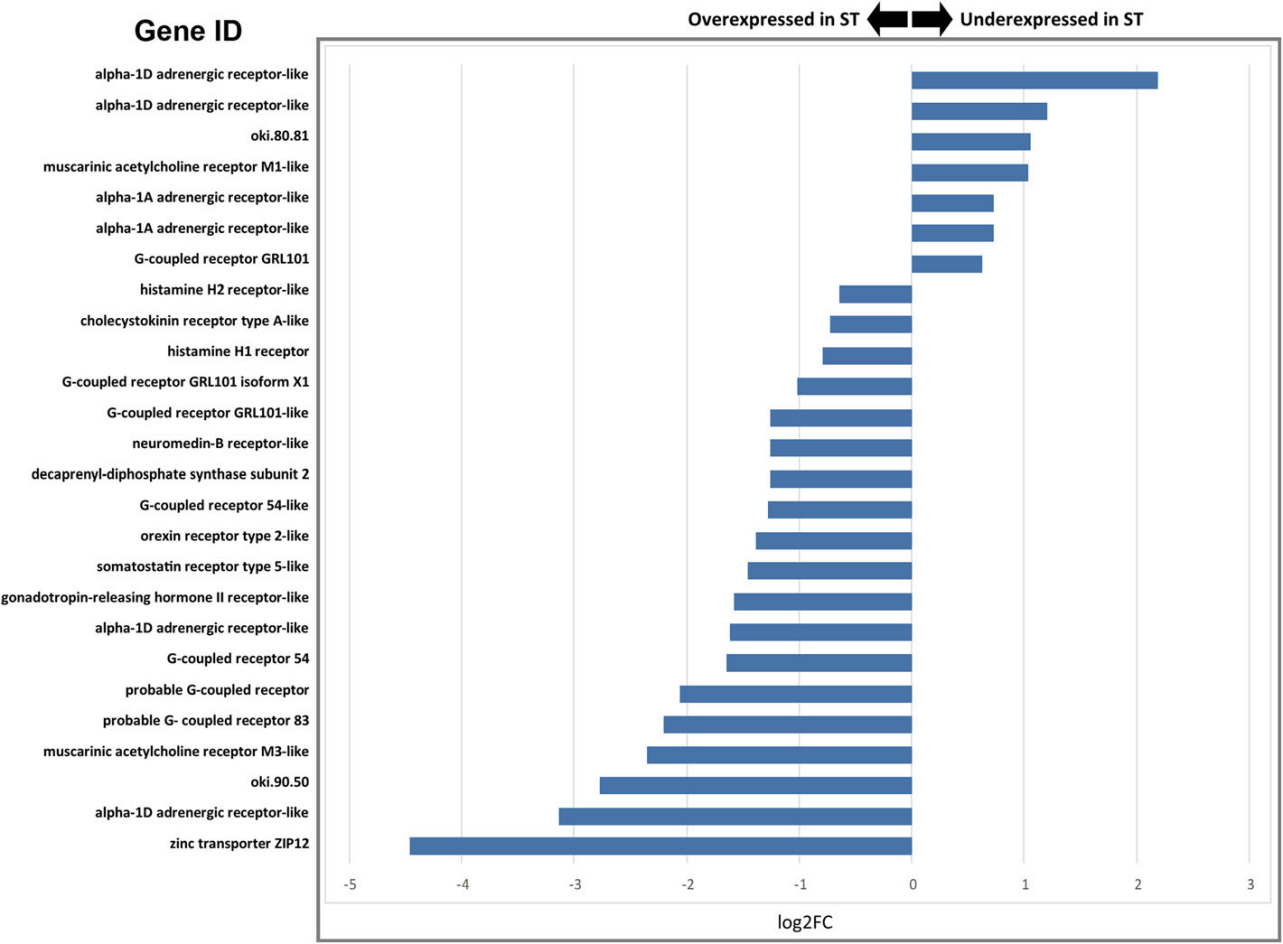
ApOR log2FC values within comparison 3 displaying under- and over-expression in COTS olfactory organs

Approximately 26 previously identified COTS ApORs (Roberts et al. 2017) were determined to be differentially expressed between three of the four sets of DEGs. In total, 24 ApORs were differentially expressed in set 3 (TF_ALL_ vs. ST_ALL_); 18 were overexpressed in ST_ALL_ and 7 were underexpressed in the same tissue (Fig. 2**)**. One ApOR was differentially expressed in set 1 (ST_F_ vs. ST_M_), a putative G protein-coupled receptor 52 (*GPCR52*), that was significantly underexpressed in ST_F_.

### Analysis of differentially expressed genes that encode for receptors

**Fig. 3:**
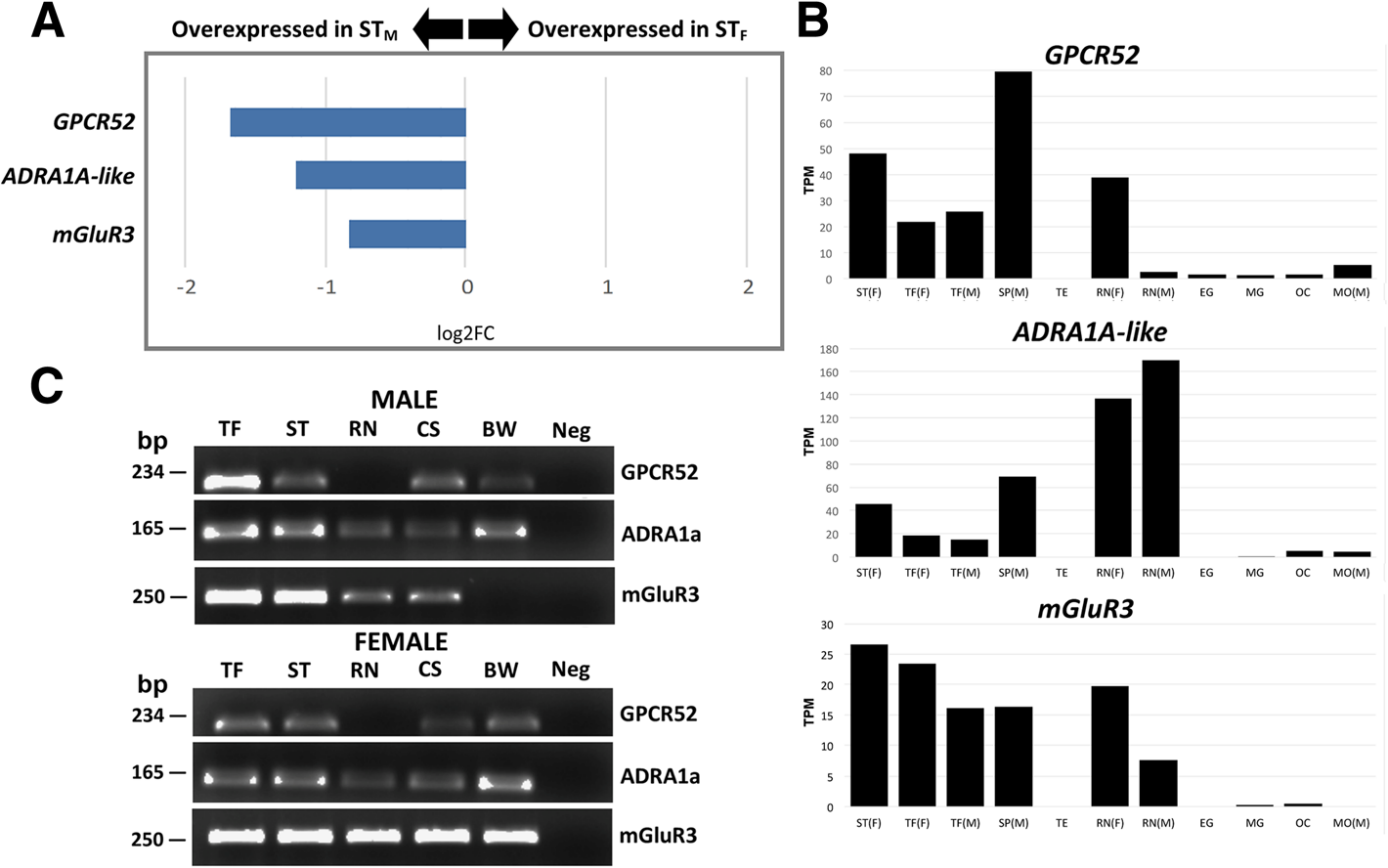
Differential expression of the three receptor genes, a G protein-coupled receptor 52 (*GPCR 52*), an Alpha-1A adrenergic receptor-like (*ADRA1A*) and a metabotropic glutamate receptor 3 (*mGluR3*), within set 1 (**a**) Log2FC gene overexpression in male COTS sensory tentacle (ST). **b** Gene expression (TPM) in multiple COTS tissues: female sensory tentacle (ST_F_), female tube foot (TF_F_), male tube foot (TF_M_) male radial nerve (RN_M_), female radial nerve (RN_F_), male spine (SP_M_), male mouth (MO_M_), testis (TE), early gastrula (EG), mid gastrula (MG), oocyte (OC). **c** Agarose gel electrophoresis of RT-PCR amplicons for 3 differentially expressed genes in multiple tissues from two pooled male and two pooled female COTS. Tissues: tube foot (TF), sensory tentacle (ST), radial nerve (RN), cardiac stomach (CS), body wall (BW), negative control (Neg). bp, base pairs

Of the DEGs containing the keyword ‘receptor’, 11 were chosen for further investigation based on their significant differential expression. Set 1 contained three of the DEGs chosen for further investigation and these three genes showed male-biased expression within the ST. This includes a G protein-coupled receptor 52 (GPCR 52), an alpha-1A adrenergic receptor-like (*ADRA1A*) and a metabotropic glutamate receptor 3 (*mGluR3*). These transcripts had log2 fold changes of − 1.6, − 1.1 and − 0.8, respectively (Fig. 3a), indicating significant overexpression in COTS male ST. When compared to expression levels in other COTS tissues, *GPCR52* still represents a relatively highly expressed gene in the ST, however, it is also highly expressed in the male spine (SP_M_), as well as RN_F_ and TF_M/F_ (Fig. 3b). RT-PCR analysis was used to determine whether these transcripts were exclusive to TF and STs, or whether they were also expressed in other tissues. All 11 DEGs chosen were successfully amplified and all of these were expressed in tissues other than the TF and ST (Fig. 3c). RT-PCR identified that *GPCR52* is also expressed in the body wall and cardiac stomach of male and female COTS. Both *ADRA1A* and *mGluR3* are expressed to varying degrees in the other tissues (Fig. 3b), which corroborate the RT-PCR results. In particular *ADRA1A* shows high expression levels within the male and female nerve tissue, as well as the male spine (SP_M_).

**Fig. 4:**
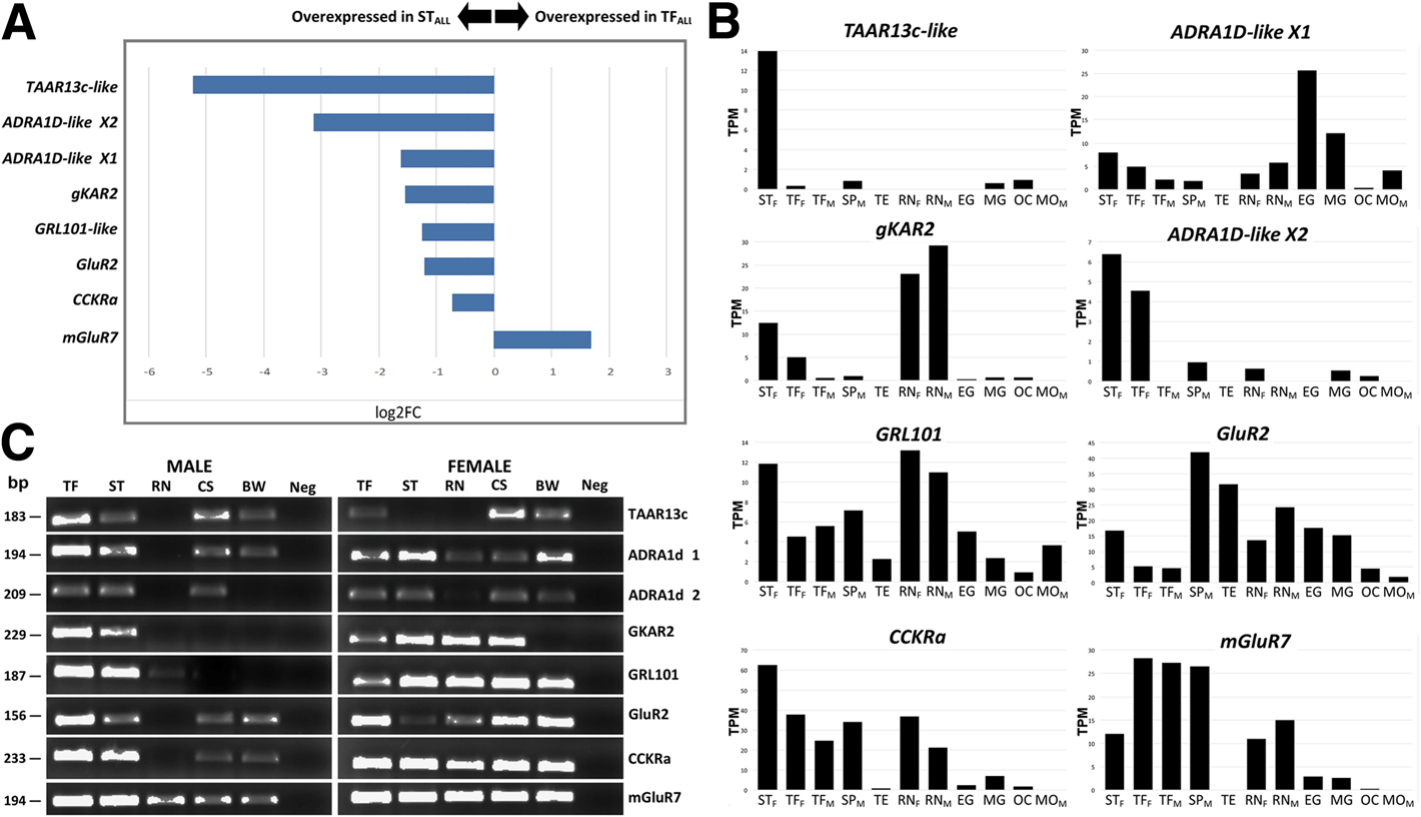
Differential gene expression of eight receptor genes within set 3, Glutamate receptor kainite 2-like (*gKAR2*), Glutamate receptor 2-like (*GluR2*), Trace amine-associated receptor 13c-like (*TAAR13c*), G protein-coupled receptor GRL101-like (*GRL101*), Cholecystokinin receptor type A-like (*CCKRa*), Metabotropic glutamate receptor 7 (*mGluR7*), and two isoforms of Alpha-1D adrenergic receptor-like (*ADRA1D-like* isoform X1 and *ADRA1D-like* isoform X2). **a** Log2FC gene overexpression in male COTS sensory tentacle (ST). **b** Gene expression in multiple COTS tissues: female sensory tentacle (ST_F_), female tube foot (TF_F_), male tube foot (TF_M_) male radial nerve (RN_M_), female radial nerve (RN_F_), male spine (SP_M_), male mouth (MO_M_), testis (TE), early gastrula (EG), mid gastrula (MG), oocyte (OC). **c** Agarose gel electrophoresis of RT-PCR amplicons for eight differentially expressed genes in multiple tissues from two pooled male and two pooled female COTS. Tissues: tube foot (TF), sensory tentacle (ST), radial nerve (RN), cardiac stomach (CS), body wall (BW), negative control (Neg). bp, base pairs

The remaining eight DEGs chosen for further investigation did not show sex-biased expression and were found in set 3 (TF_ALL_ vs. ST_ALL_), as this comparison showed the highest number of differentially expressed receptors. Of these DEGs, seven were overexpressed in ST_ALL_ and one was overexpressed in TF_ALL_ (Fig. 4a). This includes transcripts annotated as glutamate receptor kainite 2-like (*gKAR2*), glutamate receptor 2-like (*GluR2*), trace amine-associated receptor 13c-like (TAAR13c), G protein-coupled receptor GRL101-like (*GRL101*), cholecystokinin receptor type A-like (*CCKRa*), metabotropic glutamate receptor 7 (*mGluR7*), and two isoforms of alpha-1D adrenergic receptor-like (*ADRA1D-like* isoform X1 and *ADRA1D-like* isoform X2). When compared to expression levels in other COTS tissues, none of these appear exclusive to the TF and ST, however, *TAAR13c* appears to have the most specific expression within the ST (Fig. 4b). Expression of *gKAR2* is highest within the nerve tissue of male and female COTS, even when compared to the sensory organs. *GluR2*, *mGluR7* and *CCKRa* also display high expression levels in the nerve of males and females, and particularly within the male spine (SP_M_). RT-PCR results show that these transcripts are also expressed in the cardiac stomach and body wall (Fig. 4c).

**Fig. 5:**
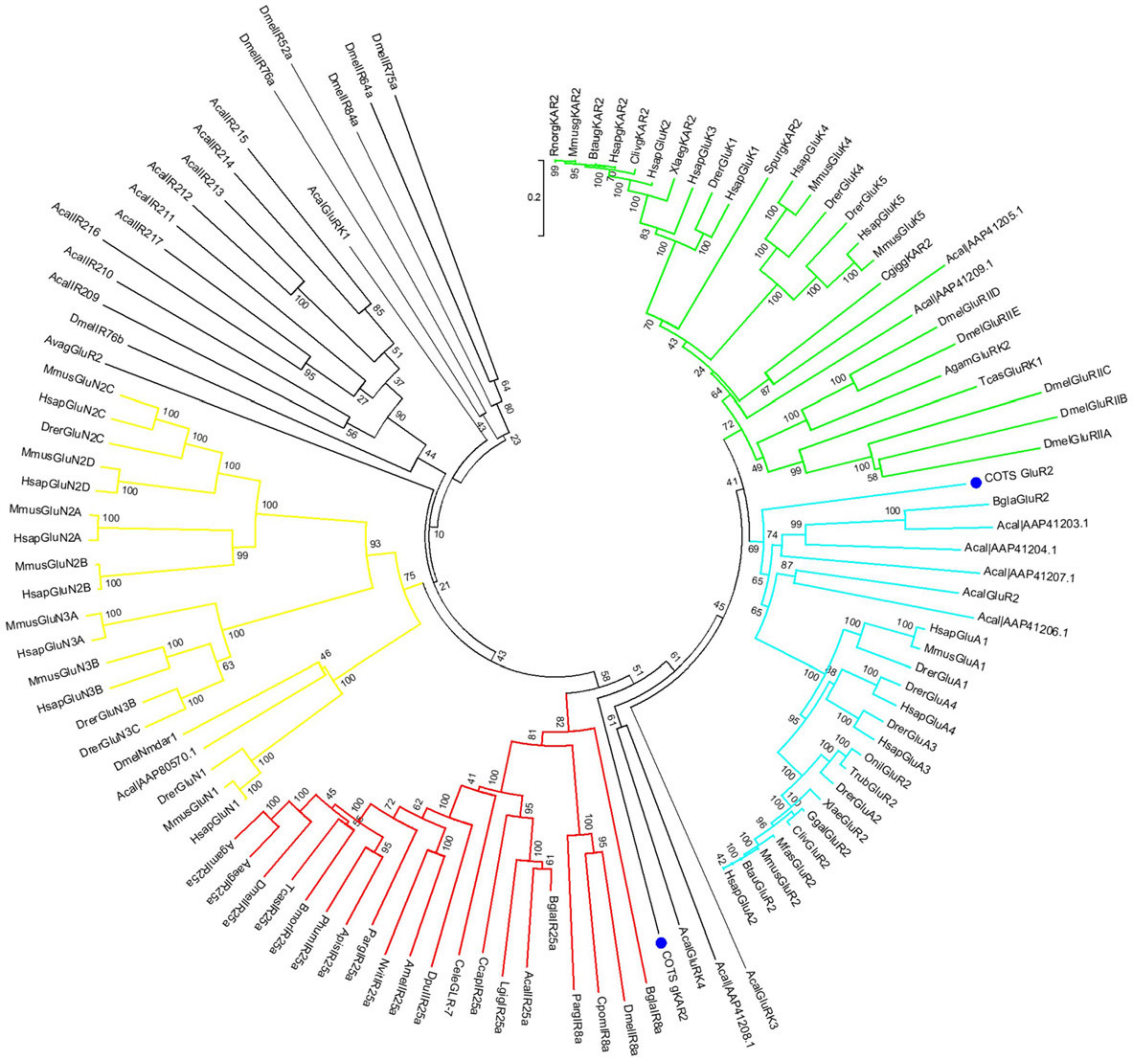
Maximum likelihood phylogenetic analysis of iGluRs and IRs. COTS transcripts annotated as gKAR2 and GluR2 are indicated with blue circles. Conserved IRs are indicated by red lines, gKAR2 (kainite receptors) are indicated by green, GluR2 (AMPA receptors) are indicated by light blue, GluN (NMDA receptors) are indicated by yellow

The transcripts annotated as *gKAR2* (a kainate receptor) and *GluR2* (a glutamate receptor) are the first receptors of this kind to be identified in a starfish. Both receptors are significantly expressed in ST_ALL_ compared to TF_ALL_ (log2FC values of − 1.5 and − 1.2, respectively) (Fig. 1a). Despite being annotated as iGluRs, their translated proteins also show high levels of similarity to the variant ionotropic receptors (IRs) that are known to be chemosensory receptors in *Drosophila melanogaster* [1], as can be observed from a multiple sequence alignment (Additional file 3: **Figure S1A**). COTS gKAR contains the N-terminal ANF domain found in the *Mus musculus* homolog, however, levels of conservation in this region are not high. Regions with the most conservation include the ligated ion-channel L-glutamate, glycine-binding site domain and the ligand-gated ion channel domain. The three key ligand-binding residues of iGluRs, arginine (R), threonine (T), and aspartate (D) or glutamate (E), do not all appear to be conserved in the COTS sequences (Additional file 3: **Figure S1B**). Phylogenetic analysis demonstrates clustering of COTS GluR2 with *Aplysia californica* and *Biomphalaria glabrata* GluR2 sequences. In contrast, COTS gKAR2 clusters with sequences from *A. californica* annotated as GluRK3 and GluRK4, as well as an unannotated *A. californica* sequence. However, they cluster near the conserved IRs and separately from the main gKAR group (Fig. 5).

Two metabotropic glutamate receptors (mGluRs; pFam family 7tm_3, PF00003) were also differentially expressed in COTS STs. Metabotropic glutamate receptor 3 (*mGluR3*) is significantly expressed in male STs compared to female STs (log2FC value of − 0.8) (Fig. 3a). When COTS mGluR3 is aligned with *M. musculus* mGluR3*,* it shows most conservation within the N-terminal ANF domain and the seven-transmembrane (7TM) region (Additional file 4: **Figure S2A**). It also displays conservation of all cysteine residues within the Nine Cysteines domain of family 3 GPCR (NCD3G) domain. Both COTS and *M. musculus* mGluR3 contain an N-terminal region ANF domain and a NCD3G region (Additional file 4: **Figure S2B**). In contrast, metabotropic glutamate receptor 7 (mGluR7) is significantly expressed in the male and female TF compared to ST, with a log2FC value of 1.6 (Fig. 4a). COTS and *M. musculus* mGluR7 also contain an N-terminal ANF domain and NCD3G region.

**Fig. 6:**
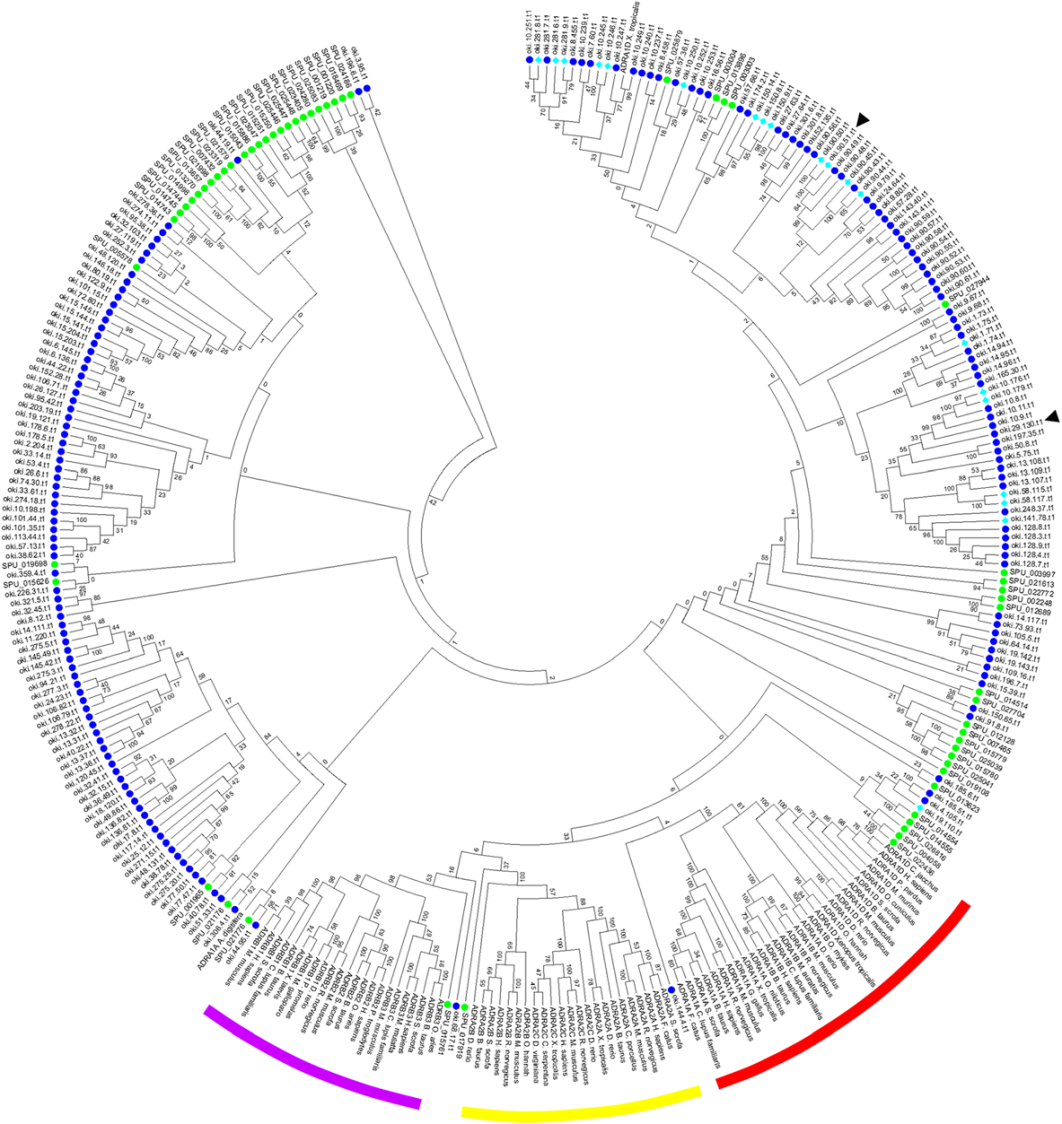
Neighbour-joining phylogenetic analysis of adrenergic receptor protein sequences (ADRs). Alpha 1 ADRs, including subfamilies 1A, 1B and 1C are represented by red lines, alpha 2 ADRs, including subfamilies 2A, 2B and 2C are represented by yellow lines, and beta ADRs, including subfamilies 1, 2 and 3 are represented by purple lines. COTS sequences are represented by dark blue circles, COTS sequences that have previously been discovered to be ApORs are represented by light blue diamonds. *Strongylocentrotus purpuratus* sequences are represented by green circles. Differentially expressed sequences in COTS are indicated by black arrows

Three adrenergic receptors (ADRs) belonging to the A17 subfamily of Pfam family 7tm_1 (PF00001) were differentially expressed in COTS sensory organs. This includes one alpha 1A adrenergic receptor (*ADRA1A*) which shows male ST-biased expression and two alpha1D adrenergic receptors (*ADRA1D-like* isoform X1 and *ADRA1D-like* isoform X2), which show ST-biased expression but no sex-biased expression. *ADRA1A* is significantly overexpressed in ST_M_ compared to ST_F_, with a log2FC value of − 1.1 (Fig. 3a). The COTS ADRA1A protein sequence has a longer N-terminus than its homolog in *M. musculus* but a truncated C-terminus (Additional file 5: **Figure S3A**). In contrast, *ADRA1D-like* isoforms X1 and X2 are both significantly expressed in the STs of both sexes (ST_ALL_) compared to the TF of both sexes (TF_ALL_), with log2FC values of − 1.6 and − 3.1, respectively (Fig. 4a). The COTS ADRA1D*-like* isoform X1 protein appears to only contain six of the seven TM domains and an extracellular C-terminus (Additional file 5: **Figure S3B**). COTS ADRA1D*-like* isoform X2 contains a complete 7TM domain, however its N- and C-termini are both shorter than its homolog in *M. musculus.* Phylogenetic analysis shows that COTS have several subfamilies of ADRs that do not cluster within any of the characterised subfamilies, including those differentially expressed (Fig. 6). Some cluster with representatives from *S. purpuratus*, however, many cluster separately from any other ADRs. Twenty previously identified ApORs are annotated as ADRs, all of which fall within the COTS-specific clusters.

The most significantly differentially expressed gene was the trace amine-associated receptor 13c-like (*TAAR13c*-like). This gene is significantly overexpressed in STs of both sexes compared to TF (ST_ALL_ vs. TF_ALL_), with a log2FC value of − 5.2 (Fig. 4a). Despite structural resemblance, the COTS TAAR13c-like protein does not display high levels of homology to its equivalent in *Danio rerio*, with only ~ 20% similarity in amino acid residues (Additional file 6: **Figure S4A**). The cholecystokinin receptor type A-like (*CCKRa*) is also significantly expressed in the STs of both sexes (ST_ALL_) when compared to the TF (TF_ALL_), with a log2FC value of − 0.7 (Fig. 4a). Despite its annotation, the COTS CCKRa protein shows significant variability from its homolog in *M. musculus*, and lacks the N-terminal CCKa_Rec_N domain (PF09193) that is characteristic of this type of receptor (Additional file 6: **Figure S4B**). *GPCR52*, which is a member of the A18 subfamily of Pfam family 7tm_1, shows ST-biased expression, being significantly overexpressed in ST_M_ when compared to ST_F_, with a log2FC value of − 1.6 (Fig. 3a). The COTS GPCR52 protein is predicted to have a relatively short N-terminus compared to *M. musculus* GPCR52, but a longer C-terminus (Additional file 6: **Figure S4C**). This protein varies in the DRY motif, which is ERY (Glu-Arg-Tyr) in COTS.

The G protein-coupled receptor GRL101-like (*GRL101*) is a member of the A10 subfamily of Pfam family 7tm_1, and displays significant expression in the ST of both sexes (ST_ALL_) when compared to the TF (TF_ALL_), with a log2FC value of − 1.2 (Fig. 4a). Significant differences exist between the COTS GRL101 and *M. musculus* GRL101 proteins. The COTS GRL101, for example, is significantly longer, and contains six LDLa motifs, compared to one LDLa motif in the *M. musculus* GRL101 (Additional file 6: **Figure S4D**). In contrast, *M. musculus* GRL101 contains more leucine-rich repeats (LRRs).

## Discussion

The main objective of this study was to investigate differences in the expression of putative chemosensory receptors in male and female Crown-of-thorns starfish (COTS; *Acanthaster* cf. *solaris*) tube feet (TF) and sensory tentacles (ST). We identify several receptor genes which display either sex- or tissue-biased expression based on their log2FC values, making them ideal candidates for further investigation in terms of functional validation and biological significance. Our results indicate significant differences in the expression of numerous 7TM receptors between the ST and TF of COTS (TF_ALL_ vs. ST_ALL_). There is also evidence of male-biased expression of three receptors within the ST (ST_M_ vs. ST_F_). In contrast, there is relatively little difference in receptor expression between male and female TF (TF_M_ vs. TF_F_). Overall, we reveal a clear difference in putative chemosensory function between the COTS TF and STs (TF_ALL_ vs. ST_ALL_), and ST of both sexes (ST_M_ vs. ST_F_). The TF_ALL_ vs. ST_ALL_ group contained the highest number of DEGs, including 295 receptor-type genes. Of these, 24 were previously identified COTS ApORs. The majority of ApORs were significantly overexpressed in the ST_ALL_ when compared to TF_ALL_. These results confirm previous research which suggested a predominantly chemosensory role for the STs in COTS [22].

Many types of receptor genes are differentially expressed in the olfactory organs of male and female COTS. In particular, 11 putative chemoreceptor genes were targeted for further analysis due to their significant differential expression pattern. Three receptor genes showed male-biased expression within the sensory tentacles of COTS: one adrenergic receptor (*ADRA1A*), a G protein-coupled receptor 52 (*GPCR52*) and a metabotropic glutamate receptor (*mGluR3*). Eight receptor genes displayed ST-biased expression but no sex-biased expression: two adrenergic receptors (*ADRA1D-like* isoform X1 and *ADRA1D-like* isoform X2), two ionotropic glutamate receptors (*gKAR2* and *GluR2*), a metabotropic glutamate receptor (*mGluR7*), a trace amine associated receptor 13c (*TAAR13c*), a cholecystokinin receptor type A (*CCKRa*) and G protein-coupled receptor (*GRL101*). While not all of these 11 genes are known to have chemosensory functions in other species, their presence within COTS olfactory organs implicates them with a likely role in chemosensation for this species.

COTS display significant overexpression of two iGluRs in their STs (ST_ALL_), *gKaR2* and *Glu2*. Both genes contain three TM domains and several conserved regions with bilaterian homologs. Both receptor genes are expressed broadly in COTS tissues. Most notably, *gKAR2* was expressed in several female tissues, but was only expressed in male TF and STs. The gKAR2 protein contains the ANF receptor domain of iGluRs, which is the extracellular ligand-binding domain [35], however, its variability in this region from the *M. musculus* homolog may be indicative of functional difference. In many invertebrates, variant iGluRs have been identified as having a chemosensory function, including *Drosophila* [1, 36], *Lepidoptera* spp. [5], *Danio rerio* [2], the water flea *Daphnia pulex* [3] and marine crustaceans such as the spiny lobster *Panulirus argus* [37] and the hermit crab *Coenobita clypeatus* [4]. More recently, glutamate receptor-like genes have been found to be critical for reproduction in mosses through sperm chemotaxis and transcriptional regulation [38]. These examples provide further support for a chemosensory role for these receptors in COTS. The COTS sequences show some conservation with both mammalian iGluRs and *Drosophila* IRs, however, they display variability in the three key amino acids which are known to bind the glutamate ligand. Phylogenetic analysis positions COTS gKAR2 separately from the gKAR2 group, however, while it bears some similarity to the IR8a and IR25a groups, it appears to have diverged after the separation of the conserved IRs from the iGluRs. Based on these results, it is likely this gene represents a possible variant IR which may be involved in chemosensation in COTS, as they are in *Drosophila* and other invertebrates.

mGluRs are class C GPCRs, originally characterised in mammalian nervous systems and having a role in neurotransmission [39]. Two types of mGluRs, *mGluR3* and *mGluR7*, were found to be differentially expressed in our comparisons. *mGluR3* shows male-biased expression within the ST, being significantly overexpressed in male STs compared to female STs, while *mGluR7* shows no sex-biased expression, being significantly overexpressed in COTS TF when compared to STs. RT-PCR demonstrated that both of these genes are also expressed in several other tissues in male and female COTS. Both are also expressed in the male spine (SP_M_) and male and female radial nerves (RN_M_ and RN_F_). Despite their original characterisation within the central nervous system of vertebrates, mGluRs have also been detected in the main olfactory bulb of *M. musculus* [40] and the olfactory organ of the sea lamprey, *Petromyzon marinus* [41]. They have also more recently been discovered to act as taste receptors in rat [42]. COTS mGluR3 and mGluR7 proteins both display conservation within the N-terminus ANF domain, which is the putative ligand-binding region, and contain all of the conserved cysteine residues within the NCD3G domain, which are known to form disulphide bridges. Given the variety of chemosensory functions of this type of receptor in other organisms, it is also likely they are involved in chemosensation in COTS.

The *ADRA1A* receptor displays male-biased expression within COTS ST, while two isoforms of the *ADRA1D-like* receptor are significantly overexpressed within the STs of COTS (ST_ALL_) compared to TF (TF_ALL_), indicating a possible olfactory role. ADRs belong to a large family that are known to control cardiovascular, respiratory and neuronal functions in humans and other vertebrates [43]. It has previously been suggested that this gene family evolved via several ancient gene duplication events in the mammalian lineage [44]. They remain relatively unexplored in invertebrates, making comparisons challenging. RT-PCR results show that they are expressed in multiple COTS tissues between males and females. Phylogenetic analysis shows distinct clustering of COTS ADRs in a manner that suggests lineage-specific expansions. Many of these were previously characterised as ApORs and cluster within the COTS genome [22]. These results confirm that COTS have a considerable expansion of ADR genes and that many of them may be involved in chemosensation.

TAARs are known to be involved in olfaction in humans, mice and other vertebrates [45, 46]. The TAAR13c receptor binds specific ligands emitted from carrion, producing an attraction/aversion response in many vertebrate species, including the zebrafish *D. rerio* [47]. In COTS, this gene shows a substantial increase in expression in STs compared to TF, the highest of any receptor investigated, however, RT-PCR shows that this gene is also expressed in several other tissues. COTS and zebrafish TAAR13c protein sequences have several conserved cysteine residues, however, there are also many regions of variability, including the characteristic DRY motif (Asp-Arg-Tyr) at the intracellular end of transmembrane helix 3. In COTS TAAR13c this motif is DRF (Asp-Arg-Phe). While this entire motif was once thought to be critical for the interaction between GPCRs and their corresponding G-proteins, it has now been established that this is not always the case [48]. The Tyr residue is the least conserved and functional studies have demonstrated that it is not involved in receptor activation [49]. If TAAR13c has a similar role in COTS as it does in zebrafish, it may be a target for biological control through interfering with COTS attraction to food stimulus such as coral.

CCKRs have been well characterised in vertebrates where they have important roles in the regulation of feeding behaviour and energy homeostasis [49]. In COTS, *CCKRa* is overexpressed in STs (ST_ALL_). *CCKRa* has also been identified in invertebrates such as *Caenorhabditis elegans. C elegans shares* significant similarity to those found in vertebrates, however, their function in invertebrates has not been confirmed [50]. The COTS CCKRa protein lacks the characteristic N-terminal domain that, in other CCKRa proteins, adopts a tertiary structure of helical turns and a disulphide cross-linked loop, and is essential for the interaction with its ligand. We speculate that its ligand-binding specificity in COTS may be different than in vertebrates, but that it likely remains involved in the regulation of feeding behaviours. Therefore, it may be an interesting target for disrupting COTS behaviours such as foraging and feeding.

*GPCR52* shows male-biased expression within the COTS ST and is an orphan receptor belonging to the rhodopsin-like family of GPCRs. In mammals it is highly expressed in the brain and inhibits dopamine signalling [51]. It has been implicated in psychosis and neurodegenerative diseases in humans and as such is a valuable target for the treatment of these conditions. In COTS, it is overexpressed in ST_M_ (ie. underexpressed in ST_F_) and RT-PCR shows expression of this gene in multiple tissues in males and females. Multiple sequence alignment between *M. musculus* and COTS GPCR52 proteins shows variability in the DRY motif at the intracellular end of transmembrane helix 3. In COTS, the Asp residue is substituted for Glu, resulting in ERY (Glu-Arg-Tyr). The Asp residue is typically conserved and forms an acidic side chain. This component is critical for regulating the activation of GPCRs and their interaction with associated G proteins. Glutamic acid is also able to form an acidic side chain however, and GPCRs with ERY motifs are still able to activate and couple to G proteins [52]. Interestingly, despite being typically highly expressed in the brains of mammals, *GPCR52* showed low expression in the radial nerve of male and female COTS (RN_M_ and RN_F_) as compared to TF and ST. This gene may have varying function between invertebrate and vertebrate phyla but its expression in COTS, particularly the male ST, implicates it in chemosensation. It may be the case that it this is a male-specific receptor which detects biological cues released from female COTS.

*GRL101* is also differentially expressed within the COTS olfactory organs, and RT-PCR shows it is expressed more so in female tissues than male tissues. This gene belongs to a family of leucine-rich repeat containing GPCRs or LGRs, including the relaxin and glycoprotein receptors originally characterised in mammals [53]. LGRs are present in a wide range of animal phyla, and a subtype of group C LGRs have been recently discovered in the purple sea urchin *S. purpuratus*, and several other invertebrates, including decapod crustaceans [54]. Based on the number of LDLa and LRR motifs, the COTS GRL101-like protein sequence bears the most homology to the type C2 LGRs, which are non-classical relaxin receptors. It has been suggested that type C2 LGRs bind insulin-like peptides (ILPs), which belong to the larger insulin superfamily of peptides [53]. Their precise function has not yet been described in non-vertebrate species, however they appear to be involved in metabolism, growth, reproduction and aging in other animals [55]. The overexpression of *GRL101* within COTS ST (ST_ALL_) suggests it may have a role in chemosensation. Based on its function in other species, it could also influence growth or reproduction.

While olfactory chemoreceptors are expressed within the sensory epithelia of specialised organs, it has been established that this is not always the case. In *Homo sapiens*, ORs are expressed in the olfactory epithelium of the vomeronasal organ, yet many of these ORs are also involved in chemosensation in tissues as diverse as muscle, sperm, kidney and the cardiovascular system (Reviewed in [24]). For example, Olfr78 binds short chain fatty acids and is expressed in the kidney where it mediates the secretion of renin [8]. Likewise, GRs that bind bitter molecules in the human tongue are also expressed in the ciliated epithelium of the lungs, where they mediate bronchodilation in response to inhaled ligands [56]. In addition, for *Drosophila* the main taste organ is the labellum, yet GRs that respond to sweet and bitter taste molecules are also found in several other tissues, including the proboscis, legs, abdomen and wings [9].These GRs are not only functional, as determined by gene knockout studies, but may be associated with specialised behaviours, including the exploration of ecological niches [9]. Based on these findings, it is not surprising that many COTS putative chemosensory receptors, such as those presented in this study, are expressed in tissues other than the TF and STs. It is also possible that the variation in expression indicates diversification of functions of these receptors.

While there is extensive literature available describing the function of ORs in terrestrial insects and vertebrates, particularly model species such as *Drosophila* and *M. musculus*, there are very few studies describing ORs in aquatic invertebrates, and even fewer in Echinoderms. This presents a considerable challenge in determining function for the number of interesting genes identified in this study. Indeed, even within vertebrate and insect lineages, ORs have a wide range of functions, many of which are unknown or not yet fully understood. Discovering which ligands bind to the ORs described in this study is essential if this is to be an avenue for the development of a biological control for COTS. Whilst homology provides a starting point, inferring the function of a differentially expressed receptor in this species based on the available literature requires further in-depth investigation that is outside the scope of this study. Thus, a significant challenge moving forward will be to elucidate the function of these receptors.

## Conclusions

These findings provide an important step forward in the development of control methods for COTS, as the identification of receptors which can be targeted by specific ligands could be the key to manipulating COTS behaviours. This could occur through interference with COTS receptor signalling or the use of chemical baits to attract COTS to specific locations for manual removal. This is the only study to describe the presence of putative variant Ionotropic Glutamate Receptors in any Echinoderm. Furthermore, they were found to be differentially expressed in the olfactory organs of COTS.

We have also discovered several other types of GPCRs such as adrenergic, metabotropic glutamate, cholecystokinin, trace-amine associated, GRL101 and GPCR52 receptors. Based on their differential expression within the olfactory organs and presence in multiple tissues, it is possible that several of these receptor types have expanded within the Echinoderm lineage and that some may be species-specific with novel ligand-binding affinity and a diverse range of functions. In addition, several receptors display male-biased expression within the STs, indicating possible reproductive significance. Many of the receptors identified in this study may be involved in key COTS behaviours, including reproduction, growth and feeding. As such, they are potential avenues for the development of novel control technologies for COTS. With further research and development in this area, there is potential to reduce the frequency and extent of COTS outbreaks and the damage they cause to coral reefs around the globe.

## Additional files

Additional file 1: **Table S1:** Gene-specific primers for 11 differentially expressed genes (DEGs) and expected amplicon sizes. (DOCX 12 kb)

Additional file 2: **File S1**. Results of differential gene expression analysis in comparisons 1–3. (XLSX 11659 kb)

Additional file 3: **Figure S1.** COTS ionotropic glutamate (iGluR) and ionotropic (IR) receptors. (**A**) Multiple sequence alignment of COTS iGluR/IR genes with homologs in *Mus musculus* (Mm gKAR2) and *Drosophila melanogaster* (Dm IR25a and Dr. IR8a). Purple line indicates region of predicted ANF terminal domain (Pfam PF01094). Blue line indicates region of predicted ligated ion-channel L-glutamate and glycine-binding site domain, annotated with its Pfam PF10613. Green line indicates region of predicted ligand-gated ion channel domain (Pfam PF00060). Key ligand-binding residues in iGluRs are shown in red boxes. **(B)** Schematic of COTS iGluR/IR with homologs in *Mus musculus* (Mm gKAR2) and *Drosophila melanogaster* (Dm IR25a). Key ligand-binding residues in iGluRs are shown in yellow with black arrows. (TIF 6374 kb)

Additional file 4: **Figure S2.** COTS metabotropic glutamate receptor 3 (mGluR3). (**A**) Multiple sequence alignment of COTS mGluR3 protein with *Mus musculus*. Purple line indicates region of predicted ANF terminal domain (Pfam PF01094). Yellow line indicates region of predicted ‘Nine Cysteines domain of family 3’ GPCR region (NCD3G). The transmembrane (TM) regions are shown with red lines, TM1-TM7. (**B**) Schematic showing COTS mGluR3 protein with its homolog in *Mus musculus*. (TIF 3613 kb)

Additional file 5: **Figure S3.** Schematic representation of COTS adrenergic receptors (ADRs). (**A**) COTS ADRA1A protein with a homolog from *Mus musculus* (**B**) Schematic representation of COTS ADRA1D-like isoform X1 and COTS ADRA1D-like isoform X2 with a homolog from *Mus musculus. (TIF 1881 kb)*

Additional file 6: **Figure S4.** Schematics showing differentially expressed sensory organ receptor proteins of COTS compared to vertebrate homologs. (**A**) TAAR13c. (**B**) CCKRa (**C**) GPCR 52 (**D**) GRL101. (TIF 1380 kb)

## Abbreviations

ADRA: Alpha adrenergic receptor
ApOR: *Acanthaster planci* putative Olfactory Receptor
CCKR: Cholecystokinin receptor
COTS: Crown-of-thorns starfish (*Acanthaster planci*)
gKAR2: Glutamate receptor kainite-like
GluR: Glutamate receptor
GPCR: G protein-coupled receptors
GRL101: G protein-coupled receptor GRL101-like
mGluR: Metabotropic glutamate receptor
TAAR: Trace amine-associated receptor
