# Supplementary material for: Putative chemosensory receptors are differentially expressed in the sensory organs of male and female crown-of-thorns starfish, Acanthaster planci
Source: BMC Genomics. 2018 Nov 29;19:853. doi: 10.1186/s12864-018-5246-0 (PMC6267866; doi:10.1186/s12864-018-5246-0)
Supplement: Supplementary file 1 — Table S1: Gene-specific primers for 11 differentially expressed genes (DEGs) and expected amplicon sizes. (DOCX 12 kb) [file 12864_2018_5246_MOESM1_ESM.docx]

| **Name** | **oki ID** | **Primer forward** | **Primer reverse** | **Size (bp)** |
| --- | --- | --- | --- | --- |
| TAAR13c | oki.15.193 | GCTAACTTGGCTGGGGAATG | AGTGAGCTTTGCCTTGGTTG | 183 |
| ADRA1a | oki.29.130 | TACCCTGTATGATCACCGCC | AGGACGAGGAAACCAGGAAG | 165 |
| ADRA1d_1 | oki.359.4 | CTCCTATGCTCCGTGTCCAT | ACCAGTCCAACACCTTTCCA | 194 |
| ADRA1d_2 | oki.90.51 | GTGCAGAGACCCAAAAGCAA | TTCTGTGAGCTCCCTGAAGG | 209 |
| GPCR52 | oki.170.40 | GCACTTATCATACCCAGCGC | TATTTCGGTAAGCTGGGCCA | 234 |
| GRL101_2 | oki.5.176 | TGTTGGACAAGGAAAGCAGC | TACCGACTATCAGCGCCATT | 200 |
| mGluR3 | oki.74.90 | TTCAGAGAGGGCAAGTTCGT | TCGGGCCCTTGATCTTTCTT | 250 |
| mGluR7 | oki.107.51 | ACCGTTATGCACACACACAC | CTGCACTTTTGACGGTCCAA | 194 |
| gKAR2 | oki.29.115 | CGCGCTTGGGATGATTAACA | CTCTTCCTCCGAGATGTCCC | 229 |
| Glu2 | oki.31.225 | TGGATGGAGTGCAAAGCCTA | AACCTAGAACGGCCGAATCA | 156 |
| CCKRa | oki.80.42 | CGTTGCTATGCTCCGCTATC | TGATCTTGAGTACGGACCGG | 233 |
