# Supplementary figures and images for: Putative chemosensory receptors are differentially expressed in the sensory organs of male and female crown-of-thorns starfish, Acanthaster planci
Source: BMC Genomics. 2018 Nov 29;19:853. doi: 10.1186/s12864-018-5246-0 (PMC6267866; doi:10.1186/s12864-018-5246-0)

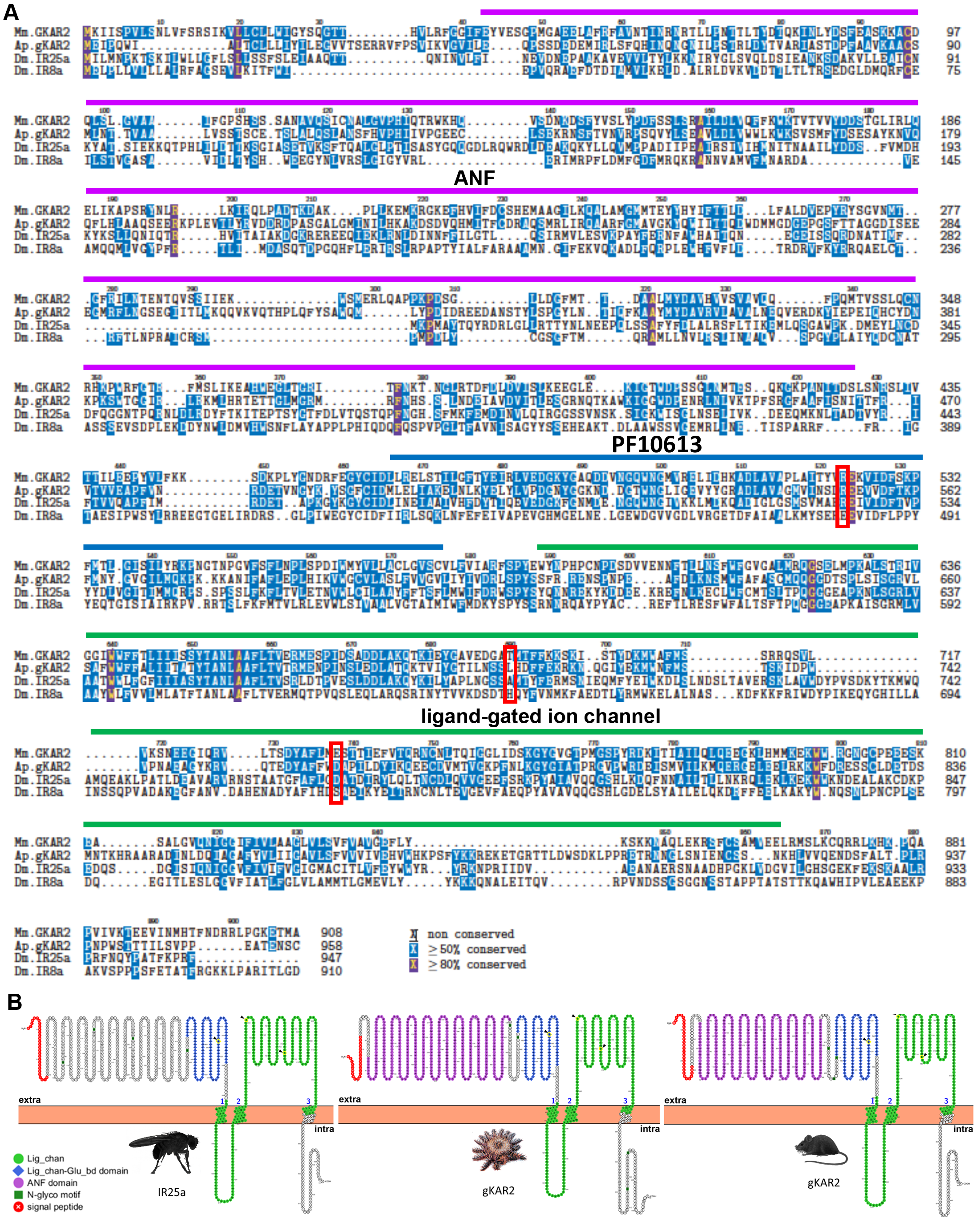

Supplement: Supplementary file 3 — Figure S1. COTS ionotropic glutamate (iGluR) and ionotropic (IR) receptors. (A) Multiple sequence alignment of COTS iGluR/IR genes with homologs in Mus musculus (Mm gKAR2) and Drosophila melanogaster (Dm IR25a and Dr. IR8a). Purple line indicates region of predicted ANF terminal domain (Pfam PF01094). Blue line indicates region of predicted ligated ion-channel L-glutamate and glycine-binding site domain, annotated with its Pfam PF10613. Green line indicates region of predicted ligand-gated ion channel domain (Pfam PF00060). Key ligand-binding residues in iGluRs are shown in red boxes. (B) Schematic of COTS iGluR/IR with homologs in Mus musculus (Mm gKAR2) and Drosophila melanogaster (Dm IR25a). Key ligand-binding residues in iGluRs are shown in yellow with black arrows. (TIF 6374 kb) [file 12864_2018_5246_MOESM3_ESM.tif]

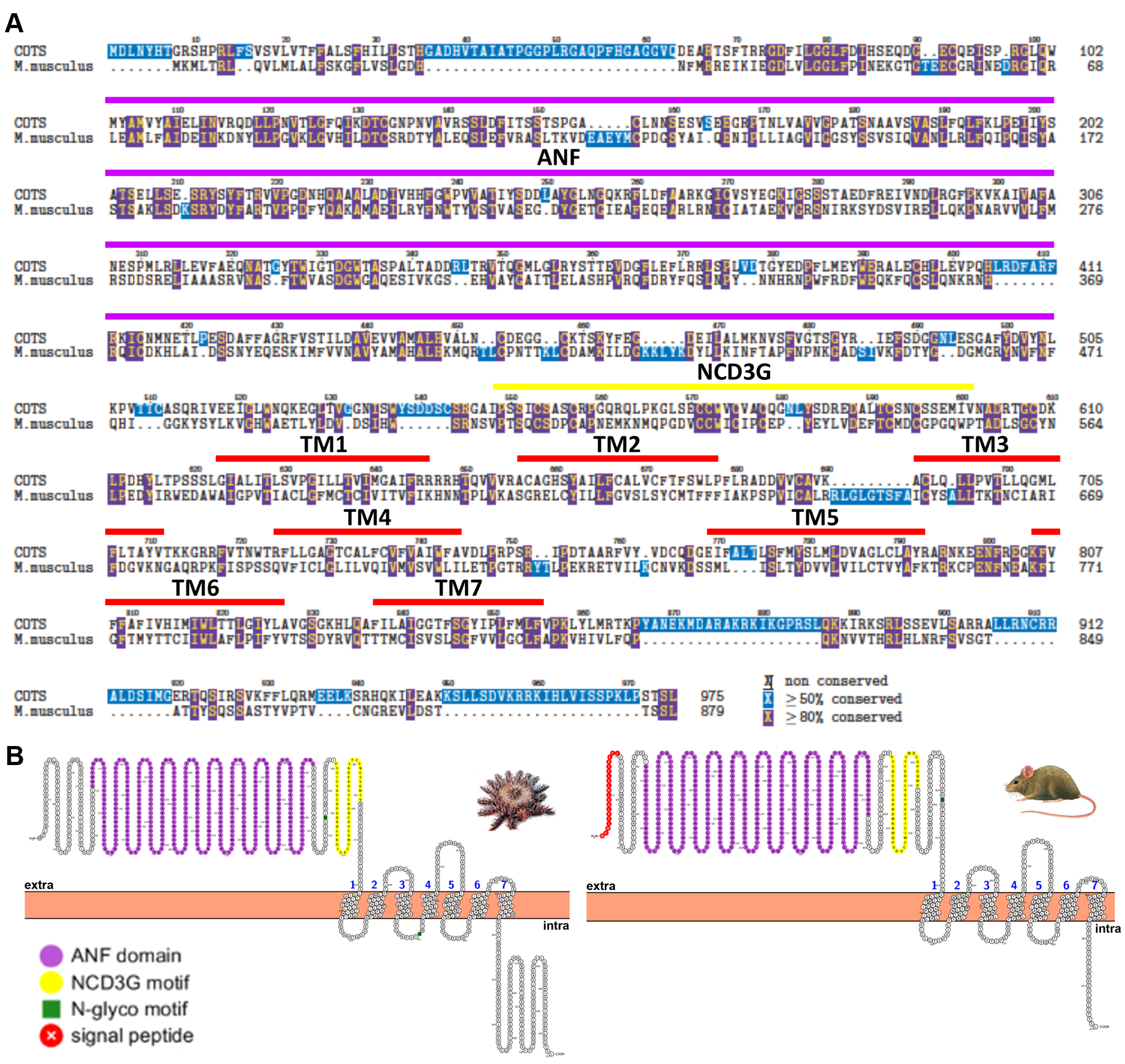

Supplement: Supplementary file 4 — Figure S2. COTS metabotropic glutamate receptor 3 (mGluR3). (A) Multiple sequence alignment of COTS mGluR3 protein with Mus musculus. Purple line indicates region of predicted ANF terminal domain (Pfam PF01094). Yellow line indicates region of predicted ‘Nine Cysteines domain of family 3’ GPCR region (NCD3G). The transmembrane (TM) regions are shown with red lines, TM1-TM7. (B) Schematic showing COTS mGluR3 protein with its homolog in Mus musculus. (TIF 3613 kb) [file 12864_2018_5246_MOESM4_ESM.tif]

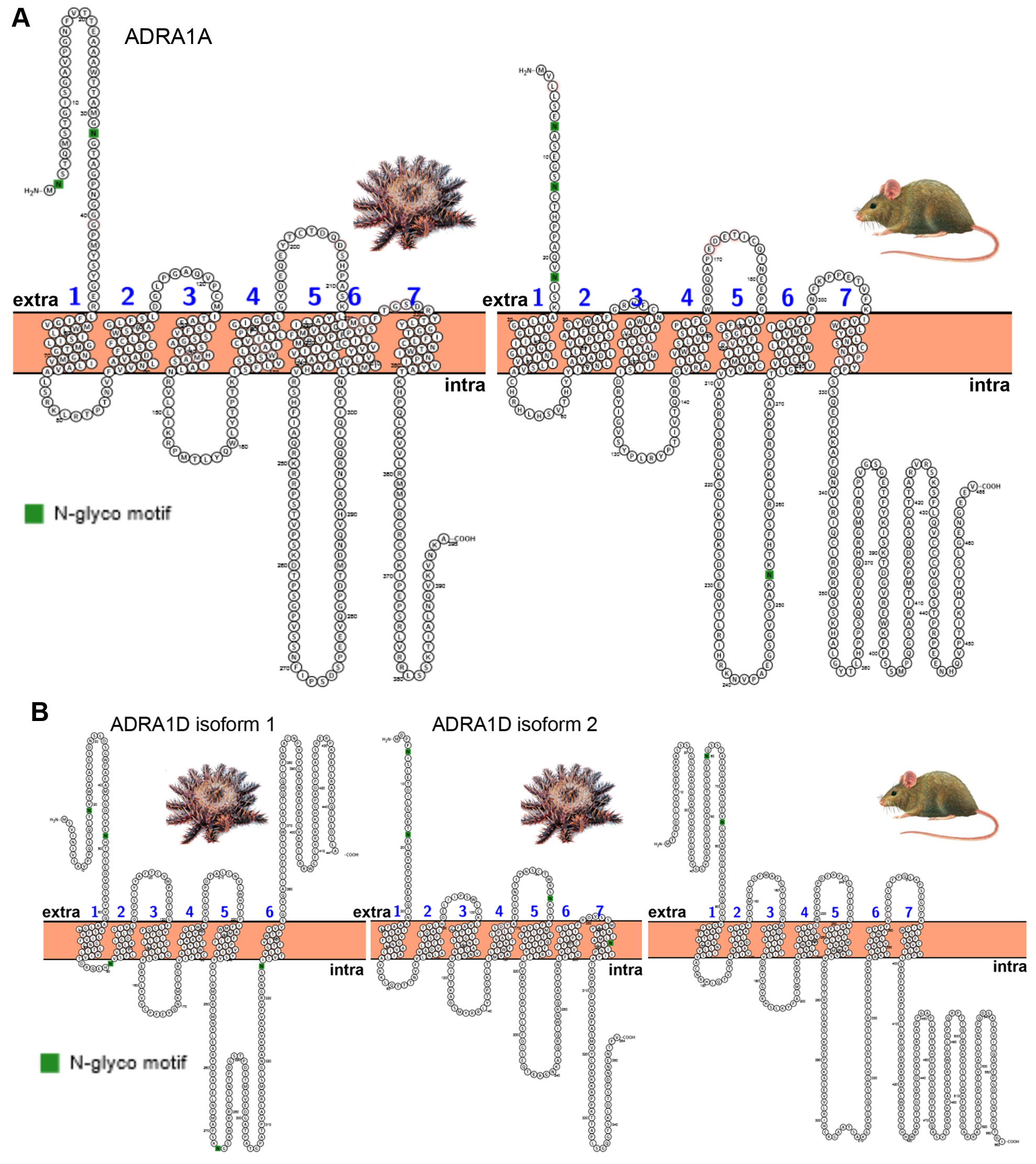

Supplement: Supplementary file 5 — Figure S3. Schematic representation of COTS adrenergic receptors (ADRs). (A) COTS ADRA1A protein with a homolog from Mus musculus (B) Schematic representation of COTS ADRA1D-like isoform X1 and COTS ADRA1D-like isoform X2 with a homolog from Mus musculus. (TIF 1881 kb) [file 12864_2018_5246_MOESM5_ESM.tif]

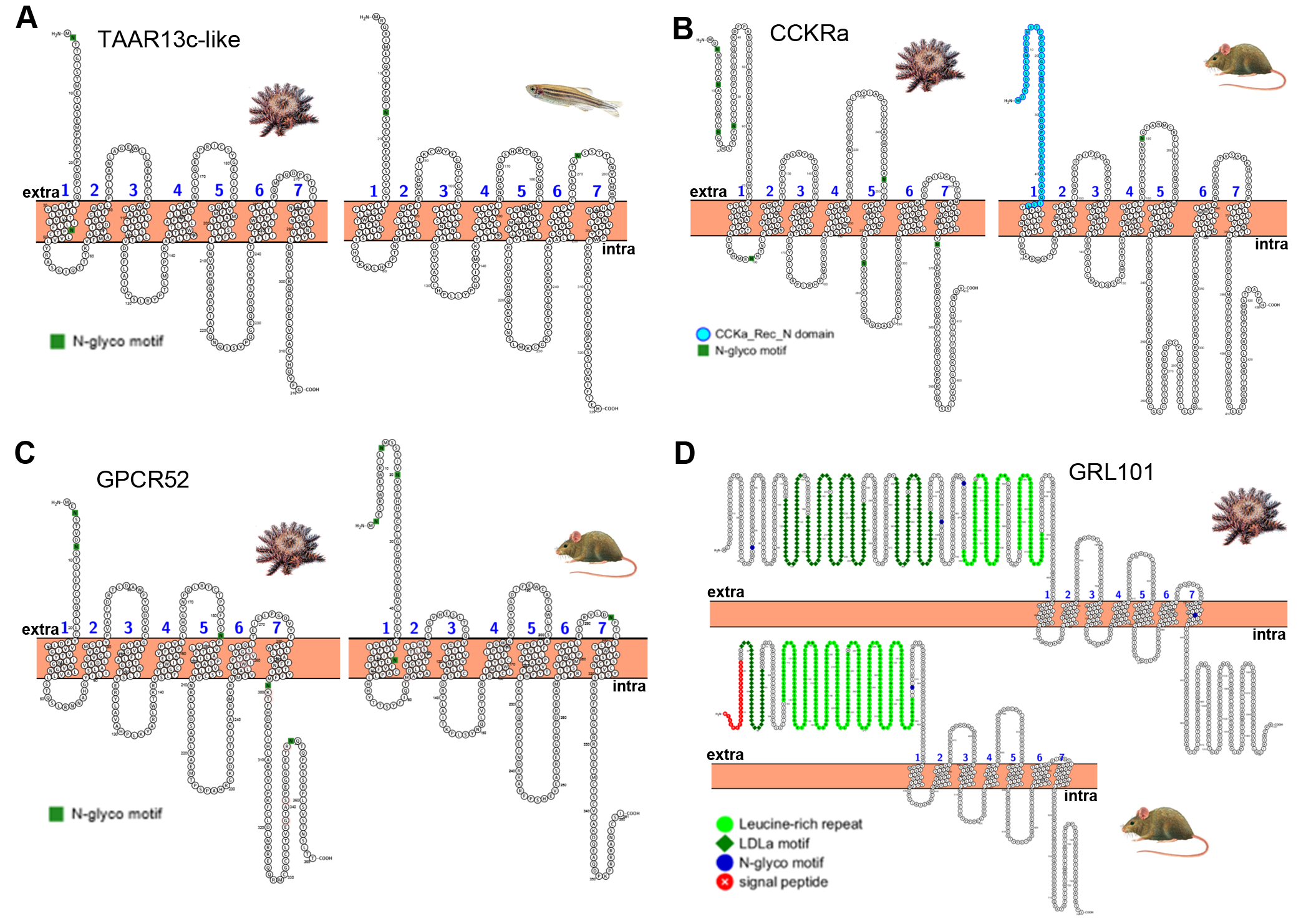

Supplement: Supplementary file 6 — Figure S4. Schematics showing differentially expressed sensory organ receptor proteins of COTS compared to vertebrate homologs. (A) TAAR13c. (B) CCKRa (C) GPCR 52 (D) GRL101. (TIF 1380 kb) [file 12864_2018_5246_MOESM6_ESM.tif]
